# No limit to maximal lifespan in humans: how to beat a 122-year-old record

**DOI:** 10.18632/oncoscience.547

**Published:** 2021-12-01

**Authors:** Mikhail V. Blagosklonny

**Affiliations:** ^1^Roswell Park Cancer Institute, Buffalo, NY 14263, USA

**Keywords:** aging, longevity, rapamycin, mTOR, centenarians, hyperfunction theory

## Abstract

Although average human life expectancy is rising, the maximum lifespan is not increasing. Leading demographers claim that human lifespan is fixed at a natural limit around 122 years. However, there is no fixed limit in animals. In animals, anti-aging interventions (dietary restrictions, rapamycin, genetic manipulations) postpone age-related diseases and thus automatically extend maximum lifespan. In humans, anti-aging interventions have not been yet implemented. Instead, by treating individual diseases, medical interventions allow a patient to live longer (despite morbidity), expanding morbidity span. In contrast, slowly aging individuals (centenarians) enter very old age in good health, but, when diseases finally develop, they do not receive thorough medical care and die fast. Although the oldest old die from age-related diseases, death certificates often list “old age”, meaning that diseases were not even diagnosed and even less treated. The concept of absolute compression of morbidity is misleading in humans (in truth, there is no other way to compress morbidity as by denying thorough medical care) and false in animals (in truth, anti-aging interventions do not condense morbidity, they postpone it). Anti-aging interventions such as rapamycin may potentially extend both healthspan and maximal lifespan in humans. Combining anti-aging medicine with cutting-edge medical care, regardless of chronological age, will extend maximal lifespan further.

## MAXIMAL LIFESPAN IN HUMANS

The life expectancy is constantly rising and median lifespan is increasing but maximum lifespan is not [1, 2]. Although the number of centenarians (100 years old or older) is doubling every ten years, maximum longevity remains the same [1]. The longest living person died in 1997 at the age of 122 and this record has not been beaten.

Therefore, it was suggested that maximum lifespan of humans is fixed and subject to natural constraints [3, 4]. Based on purely demographic data, natural limit to human life span was estimated to be between 115 years [3, 4] and 126 years [5]. Furthermore, Olshansky et al. believe that the absence of people, who are older than 122, is the evidence for why there are limits to human longevity [6].

It was suggested that longevity records cannot be overcome unless a scientific breakthrough in delaying aging would happen [7]. First, such scientific breakthroughs are happening now and drugs that slow down aging are becoming available (see for ref. [8]). Yet, these drugs have not yet been employed in a sufficient number of humans for a sufficiently long period of time to make demographic impact. This breakthrough will eventually break the lifespan record. However, such a breakthrough is not even necessary. **A mere application of standard medical care to centenarians, as rigorously as to younger adults, would probably extend lifespan beyond 122, even without the need of a scientific breakthrough. **We will discuss here that **an increase of average lifespan without maximal lifespan is happening because advanced medical interventions are available for everyone except the oldest old, exactly those who may live longer than 122, if treated**. While a thirty-year old patient with heart disease may become a candidate for heart transplantation, it would be ridiculous even to mention heart transplantation for a supercentenarian. In other words, life-extending care is not available (usually with best intentions) exclusively and specifically to those who can beat the 122 lifespan record. Furthermore, since their death certificates state “old age” instead of a specific disease, most centenarians do not receive treatment but even a diagnosis [9, 10]. As we will discuss, this explains why the 122 year record is not broken despite the absence of any biological constraints.

## HEALTHSPAN, LIFESPAN, MORBIDITY, EXPANDED AND CONDENSED MORBIDITY: GENERAL NOTION

Humans and other animals, including *C elegans*, die from age-related diseases. *C elegans* suffer from quasi-programmed age-related diseases such as intestinal atrophy, yolky lipid accumulation and teratoma-like tumors [11–14]. Most common age-related diseases and pathologies in humans are atherosclerosis and cardiovascular disease, cancer, obesity and type 2 diabetes, hypertension, Alzheimer’s and neurodegenerative diseases, osteoporosis, osteoarthritis, sarcopenia and others. In humans, age-related diseases are late manifestations of aging [15]. Those humans, who age slower, develop diseases later in life and live longer. Slowly aging centenarians do live longer, because age-related diseases are delayed. For example, centenarians are protected against cancer [16].

**Figure 1:**
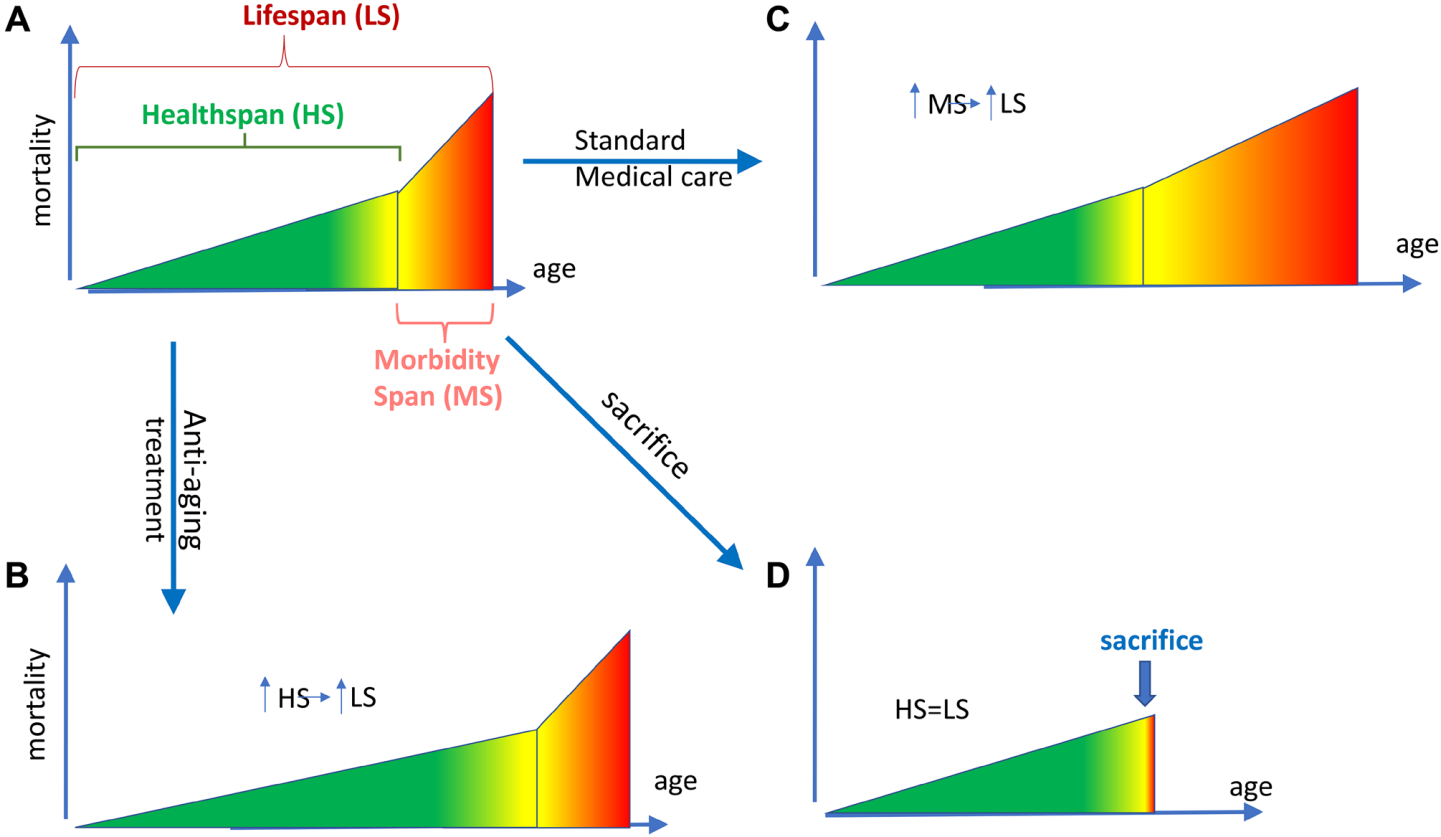
Two ways to increase lifespan and one way to shorten it. (**A**) Defining healthspan (HS), morbidity span (MS) and lifespan (LS). Healthspan (green) is a period of life without age-related diseases. Morbidity span (red), a period with diseases, culminates in death. Healthspan (HS) plus Morbidity Span (MS) constitute lifespan (LS). The border between HS and MS is somewhat arbitrary (yellow). With age, morbidity increases, reaching the death threshold. (**B**) Anti-aging interventions extend lifespan by increasing healthspan. In animals, anti-aging interventions delay the onset of age-related diseases. An extended healthspan (measured by the absence of life-limiting diseases) automatically increases lifespan because morbidity span is unchanged. When aging is slowed, lifespan is expanded. (**C**) In humans, anti-aging interventions have not yet been employed. Life extension is achieved by expansion of the morbidity span by medical care. (**D**) An animal can be sacrificed during morbidity phase to prevent animal suffering. (In fact, current rules require animal sacrifice at certain conditions, like large and ulcerous tumors). This leads to artificial compression of morbidity.

Healthspan is a period of healthy life, which ends with the onset of age-related diseases, followed by morbidity span (Figure 1A). Although healthspan is difficult to measure precisely, it’s a useful abstraction [17]. Healthspan (HS) + morbidity span (MS) = lifespan (LS). An increase in healthspan increases lifespan (Figure 1B). (Note: For example, in centenarians the onset of diseases is delayed and therefore they live longer. They could live even longer, if they would receive thorough medical care when diseases develop.) Standard medical interventions treat each disease separately and thus extend lifespan by increasing morbidity span (Figure 1C).

**Morbidity not only can be expanded but also compressed. In mice, morbidity span can be intentionally compressed by sacrificing them (Figure 1D). **When an advanced disease is detected (e.g., tumor above certain size), animals are sacrificed to prevent their suffering.

As we will discuss later, **in the oldest humans, morbidity is “relatively compressed”, not by active sacrifice, but by passive “denying” (often by patients themselves) of cutting-edge medical care, otherwise available for the younger patients. **(Note: I use the word “denying” for brevity: in many cases, patients themselves do not want aggressive medical interventions).** This gives the impression that morbidity is compressed in centenarians, although it is simply expanded in everybody else, except centenarians **(relative compression).

## ABSOLUTE COMPRESSION OF MORBIDITY IS A MISLEADING CONCEPT

In the last 20 years, numerous drugs gained popularity as potential anti-aging drugs, including flavonoids, NAD boosters and senolytics. Disappointingly, most of them failed to extend lifespan in animals (see for references [8]). Therefore, it become acceptable that anti-aging drugs should not make animals live longer but only healthier. A new paradigm proposes compression of morbidity without extension of lifespan. Thus, Olshansky “welcome … a compression of morbidity, but only a marginal further increase in life expectancy,” [6].

The question is how then humans will die, if not from diseases. From good health? The idea of dying in good health is based on a misconception that age-related diseases and aging are not one and the same. It is erroneously believed that an animal including humans may die from either aging or age-related diseases. In reality, they always die from deadly manifestations of aging (age-related diseases). The oldest old such as super-centenarians die from age-related diseases [9, 10].

If so, to compress morbidity, diseases must develop with astronomical speed. Would cancer grow in a matter of minutes instead of months? No, cancer tend to grow slower in the elderly [18]. Or, for example, development of atherosclerosis begins in childhood [19] and it cannot be compressed in months.

**Figure 2:**
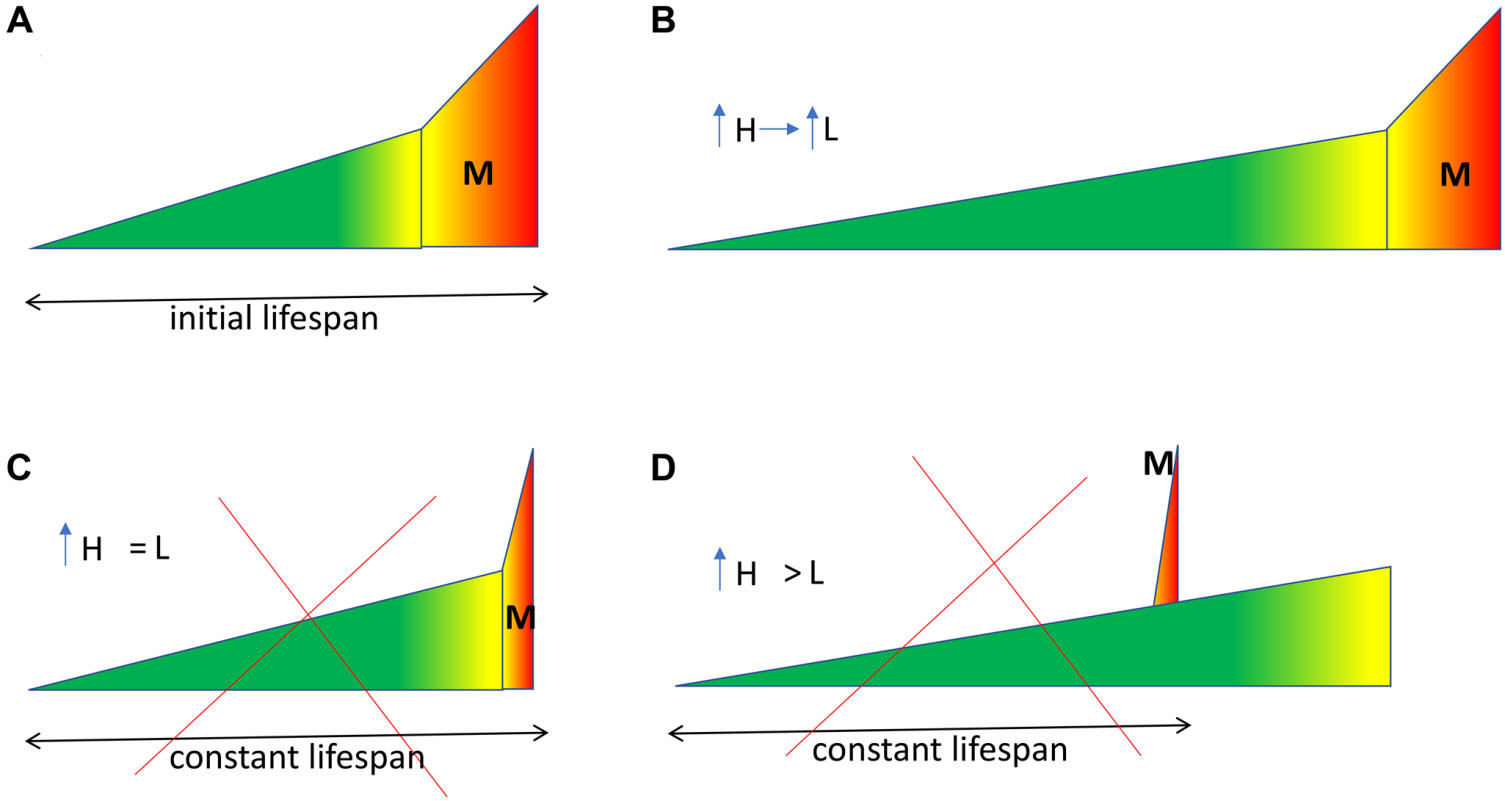
The problem of absolute compression of morbidity concept. See Figure 1A for symbol explanation. M morbidity span. (**A**) Initial lifespan. An untreated animal. (**B**) Anti-aging intervention. Morbidity span is constant, healthspan and lifespan are expanded. So, morbidity “compressed” only relatively, not absolutely. (**C**, **D**) Fictional. Lifespan is kept constant, regardless of healthspan extension. A progressively expanded healthspan, with constant lifespan, is bound to absurd.

Morbidity span does not change, when healthspan is extended (Figure 2A, 2B). It cannot be absolutely compressed (only relatively) by extending healthspan, unless we sacrifice an animal at the onset of diseases (Figure 2A, 2C). **Humans can be “sacrificed” unintentionally by avoiding aggressive treatment in the oldest old **(for whatever good reasons). When aging is slowing down even further, while progressively increasing healthspan, then lifespan cannot be kept constant, even if we sacrifice an animal. Consider healthspan extended beyond initial lifespan (Figure 2A, 2D). Then to keep lifespan constant, an animal must die before the end of healthspan (Figure 2D). **Healthspan longer than lifespan is absurd.**

When healthspan is extended, then morbidity becomes only relatively compressed, rather than absolutely [20, 21].

## “HEALTHSPAN WITHOUT LIFESPAN” IS NOT GENUINE HEALTHSPAN

How then have studies in mice demonstrated the extension of healthspan without extension of lifespan. One possibility is that, in those studies, real healthspan was not measured. Instead, artificial healthspan was measured using arbitrary biomarkers. Genuine healthspan is measured by the absence of deadly age-related diseases, such as cancer in mice. In fact, interventions that extend lifespan such as rapamycin, delay cancer in mice, extending real healthspan [22–24].

There are life-limiting age-related diseases such as cancer and non-life-limiting age-related conditions such as grey hair. (Noteworthy, in humans, boldness, grey hair and facial wrinkles do not predict mortality [25, 26]. Although not life-limiting, grey hair is often used to measure health in mice.

If lifespan is not increased, despite an increase in lifespan, then such “health markers” are not life-limiting by definition. When aging is slowed down, diseases are delayed and slowly aging individuals must live longer, exactly because diseases are delayed. Naturally-slowly aging individuals are centenarians.

## CENTENARIANS

Centenarians can be divided into survivors and delayers/escapers [27]. Survivors develop age-related diseases earlier in life but survived to the age of 100 due to medical treatments. In other words, they are not natural centenarians, but reach the 100-year-old threshold with the help of thorough medical care.

**Figure 3:**
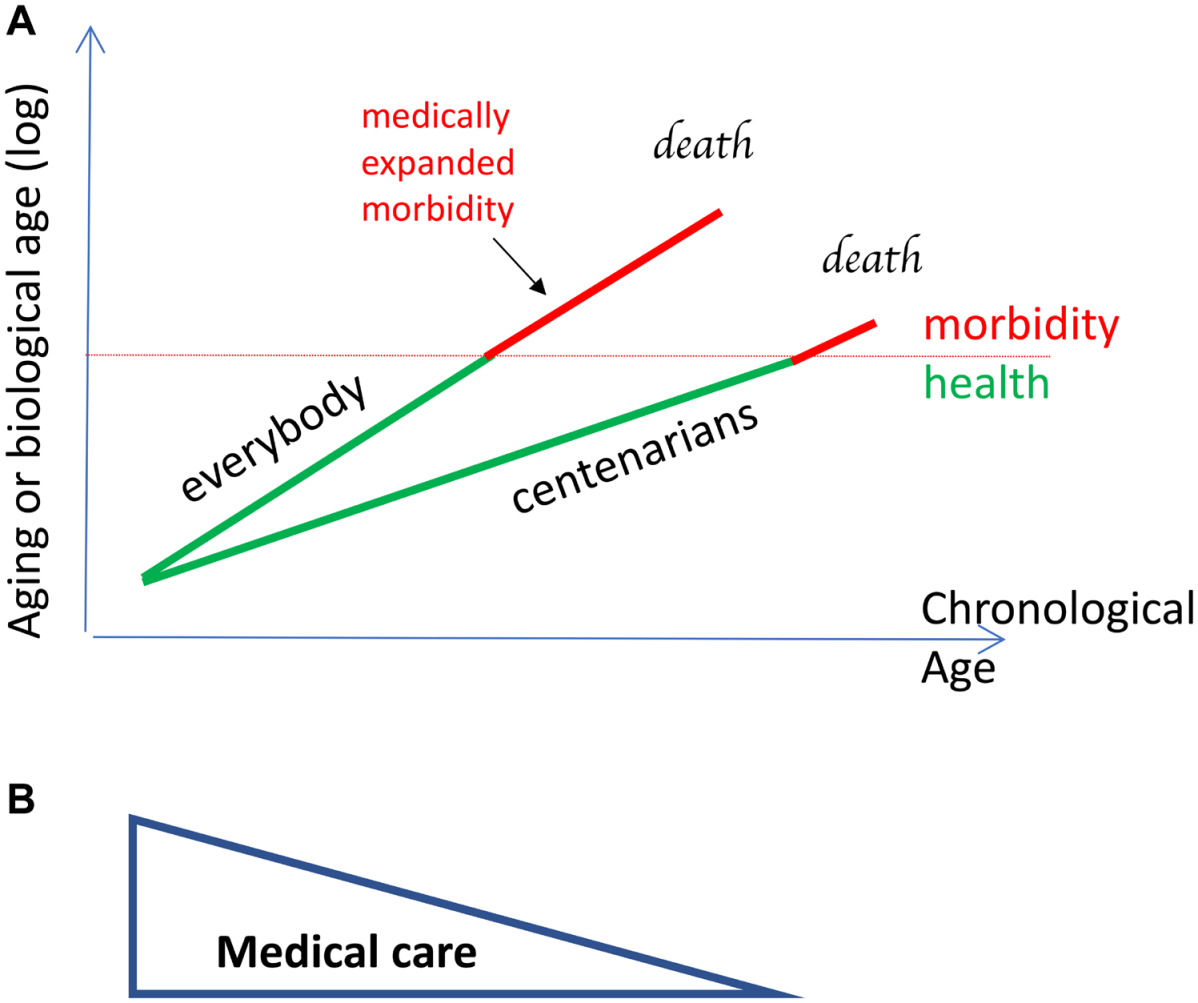
Centenarians age slow and develop diseases late, but do not receive medical care when diseases develop. (**A**) Aging and biological age are log of chronological age because mortality increases exponentially. Centenarians age slower and their biological age is less than chronological age. The availability of medical care decreases with chronological age and centenarians receive less care because morbidity develops at a higher chronological age. So, morbidity span is not medically expanded in centenarians, creating the illusion of compression, relatively to everyone else. It is a relative compression. Green lines: healthspan. Red lines: morbidity span. Note: morbidity starts at the same biological age for both groups. (**B**) Reverse relationship between chronological age and health care.

In contrast, natural (slowly-aging) centenarians (delayers/escapers), are characterized by a slow rate of aging and delayed onset of age-related diseases (Figure 3A). Biological age is lower than chronological age in natural centenarians (Figure 3A). We will discuss only natural (slowly aging) centenarians.

In centenarians, life-limiting diseases (e.g., cardiovascular diseases, cancer [16]) but not necessarily non-life limiting diseases/conditions (e.g, wrinkles, grey hair) are delayed [25, 26].

Centenarians, especially supercentenarians, reach old age in good health, indicating slow aging [16, 28–30]. Then, however, they deteriorate fast [1]. In supercentenarians, morbidity is especially compressed [28, 29, 31]. Why?

## CENTENARIANS DO NOT RECEIVE MEDICAL CARE

The cost of end-of-life healthcare for centenarians is substantially less than that for non-centenarians [32]. Ironically, these data are misinterpreted as if centenarians do not need medical care, but the data actually mean that they do not receive medical care. Centenarians use less healthcare services than octogenarians (80–89 yo) and nonagenarians (90–99 yo) [33].

The lessons of the COVID-19 pandemic are revealing. In nursing homes, despite a higher mortality rate of centenarians, their hospitalization rate was much lower than that of younger patients [34].

As another example, hip replacement is very rarely done for centenarians [35, 36]. However, it was found that hip replacement should be performed in centenarians [35, 36], and successful hip surgery in a 107-year-old patient was described [36]. The centenarians well tolerate joint arthroplasty, spine surgery, laparoscopic cholecystectomy, aortic valve repair, and other vascular procedures. It was concluded that the oldest old should not be denied these medical procedures “on the basis of chronologic age, and they deserve equal resources as younger people,” [37].

Adequate cancer treatment is often not available for elderly patients because of their advanced chronological age [38–40]. This may compress morbidity compared to the younger patients receiving adequate treatment [38–40].

In one study, 62.7% of patients older than 80 years with lung cancer (stage III) did not receive any cancer-directed care. However, survival was prolonged in patients treated with chemoradiation [38]. Older patients with advanced lung cancer have lower chances of receiving cancer treatments, compared to younger patients, even when their other morbidity and performance are equivalent [40]. Pham et al. called this **“treatment nihilism towards elderly patients” **[40]. With an increasing age, costs of medical care decreased drastically, outpatient treatment with pain medications was decreased too [39]. Walter et al. suggested that older patients are at risk of insufficient treatment [39].

The older the person, the fewer medical interventions are provided (Figure 3B). One reason is an erroneous belief that, although humans and other animals die from age-related diseases, the very old humans die of old age. In reality, everyone dies from age-related diseases and supercentenarians are not exception [10].

The second reason why the oldest old do not receive medical care is that physicians may consider it “cruel” to aggressively treat fragile oldest old, who are believed destined to die of old age “peacefully”.

The third reason is that some oldest old themselves may not want medical care.

The fourth reason is that some resources are limited. For example, heart transplants are limited by the number of donor hearts available. It is accepted that finite health care resources should be spent on the youngest patients. Even hip replacement may be denied to centenarians solely on the basis of age [35].

Whatever the reason, centenarians cannot beat the 122-year-old barrier.

In centenarians, slow aging delays diseases to the age when medical care become almost nonexisting. The diagnosis is usually unknown even after death (“old age” as diagnosis). The evidence of good health is usually based on self- and proxy reports of the age of onset of age-related diseases [30]. As shown by Berzlanovich, although most centenarians were believed to be absolutely healthy before death, they died from typical age-related diseases in 100% of the cases examined [9, 10]. In addition, these centenarians suffered from several comorbidities, which were not judged to be the cause of death [10].

**Figure 4:**
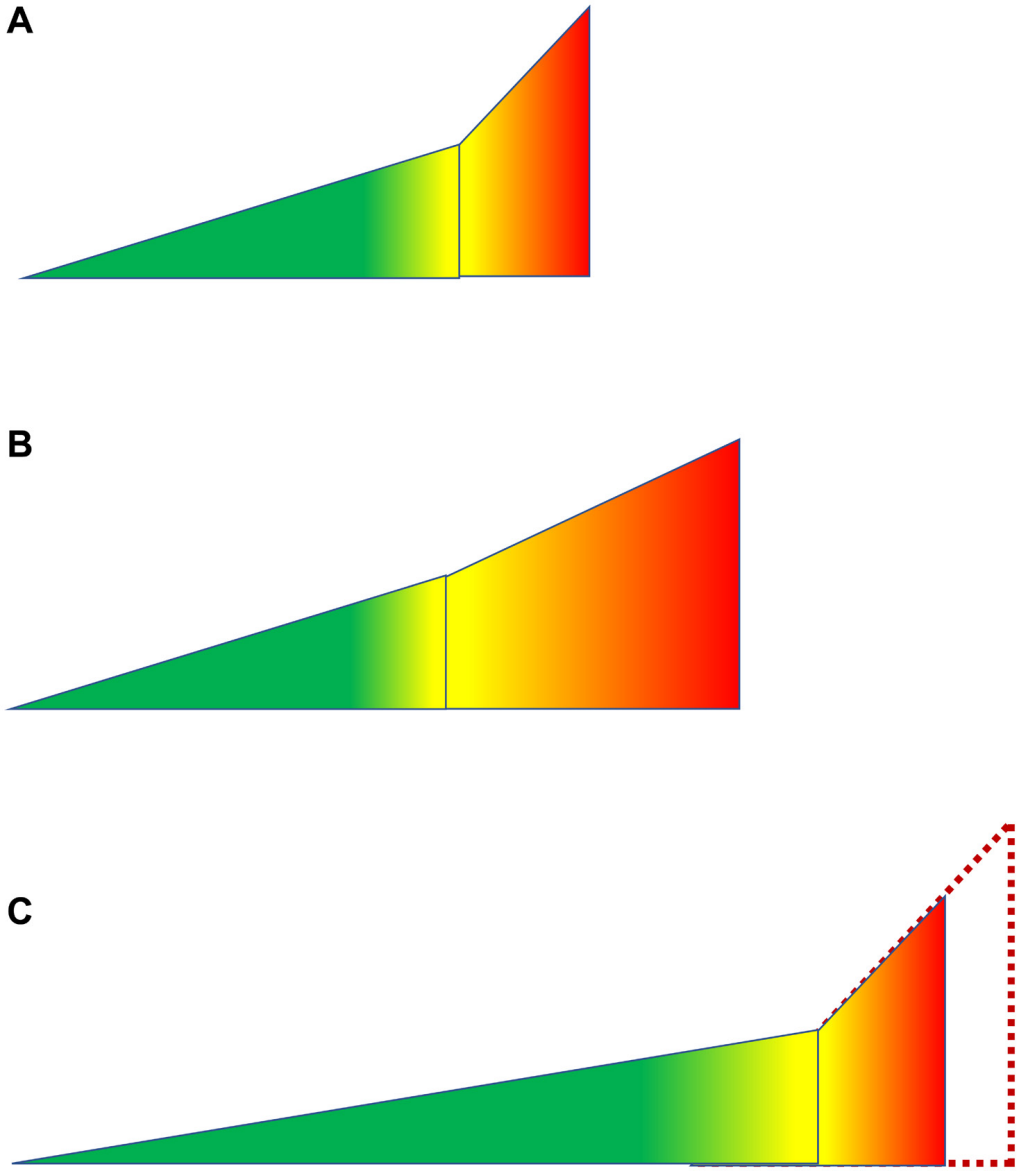
Illusion of compressed morbidity in people with exceptional longevity. See Figure 1A for symbols. (**A**) Everyone (an average person). No health care. (**B**) Everyone (an average person). Current health care. Improvements in health care drive lifespan by increasing morbidity span. (**C**) Exceptional longevity due to slow aging and extended healthspan. Because diseases develop at older chronological age, they are not diagnosed or treated. The morbidity span is comparable to those shown in panel A. So, compression is relative to panel B. If health care would be applied according to biological, not chronological age, then lifespan would be extended: dashed line.

In centenarians, morbidity is relatively compressed because it is not expanded compared to rapidly aging individuals, who develop diseases at younger age and get thorough treatment (Figure 4).

## FIRST CONCLUSION

This article presents a simple explanation for why average lifespan is constantly increasing, while maximum lifespan is not. Constant advancements and improvements of medicine care lead to increased morbidity span and thus lifespan in everybody except the oldest old, because of the reverse relationship between chronological age and medical care. The later the onset of morbidity and thus, a longer lifespan, the less medical attention and care one receives. Consider an extreme hypothetical example: a billionaire supercentenarian attempting to have a heart transplant and willing to pay billions. That probably will be illegal. This can explain in part why “there has not been any improvement in mortality amongst centenarians in the past 30 years.” [2].

Improvement can easily be made by not denying medical care to them (Figure 4). Then supercentenarians may beat the 122 record (Figure 4). (Note: I use the word “denying” for brevity: in many cases, patients themselves do not want aggressive medical interventions).

## PREDICTION: GENDER GAP WILL BE SHRINKING

If people with early morbidity and low life expectancy benefit the most from continuous improvement of medical care, men must benefit more than women. Because Men live shorter lives than women and because men develop deadly diseases at an earlier chronological age. In theory, chronological age of older women prevents them from receiving the same care as younger men.

In fact, the gender gap is closing in all countries, driven by improvement of medical care [41–45].

The life expectancy gender gap is closing fast: it would narrow to 1.9 years by 2030. https://www.bbc.com/news/health-32512351

https://www.independent.co.uk/life-style/life-expectancy-men-women-same-2030-uk-deprived-areas-research-ilc-a8276131.html

## HOW TO DECREASE “RATE OF AGING”

A group of leading demographists co-authored a study in primates, “implying constraints on how much the human rate of aging can be slowed” [46]. The study however does not include experiments attempting to increase lifespan in monkeys. The hypothetical conclusion at the end of the discussion is: “improvements in the environment are unlikely to translate into a substantial reduction in the rate of ageing in humans” [46].

Improvements in the environment cannot change the rate of aging. But rapamycin can.

In a large study by Harrison et al. 2009, rapamycin given to mice late in life extended lifespan of the last survivors in genetically heterogeneous mice [47]. As published in 2010 by Anisimov et al., parameter α of the Gompertz model, which stands for the rate of aging, was 1.8 times lower in rapamycin-treated mice compared with control mice [24]. Rapamycin extended healthspan (cancer-free survival), increased mean and maximal lifespan in these cancer-prone mice [24]. Notably, the increase in mean lifespan was relatively modest but increase in maximal lifespan was significant (12.4%) [24]. In female 129/Sv mice, rapamycin also decreased rate of aging, increased lifespan in the last survivors [48]. Thus, 22.9% of rapamycin-treated mice survived the age of death of the last mouse in the control group [48]. In some mutant short-lived mice, rapamycin triples maximum lifespan [49]. The higher the dose of rapamycin, the longer lifespan [50–52].

Maximal lifespan is not fixed in primates either [53]. In gray mouse lemurs, 30% calorie restriction (CR) diet increases healthspan, increasing maximum lifespan by 22% (13.8 years in the CR group vs. 11.3 years in the control group) [53].

## UNLIMITING “LIFESPAN LIMIT” FOR EVERYONE

As we discussed, the lifespan of slowly aging centenarians can be extended by providing them adequate medical care. But can an average person beat the 122-year-old record?

**Figure 5:**
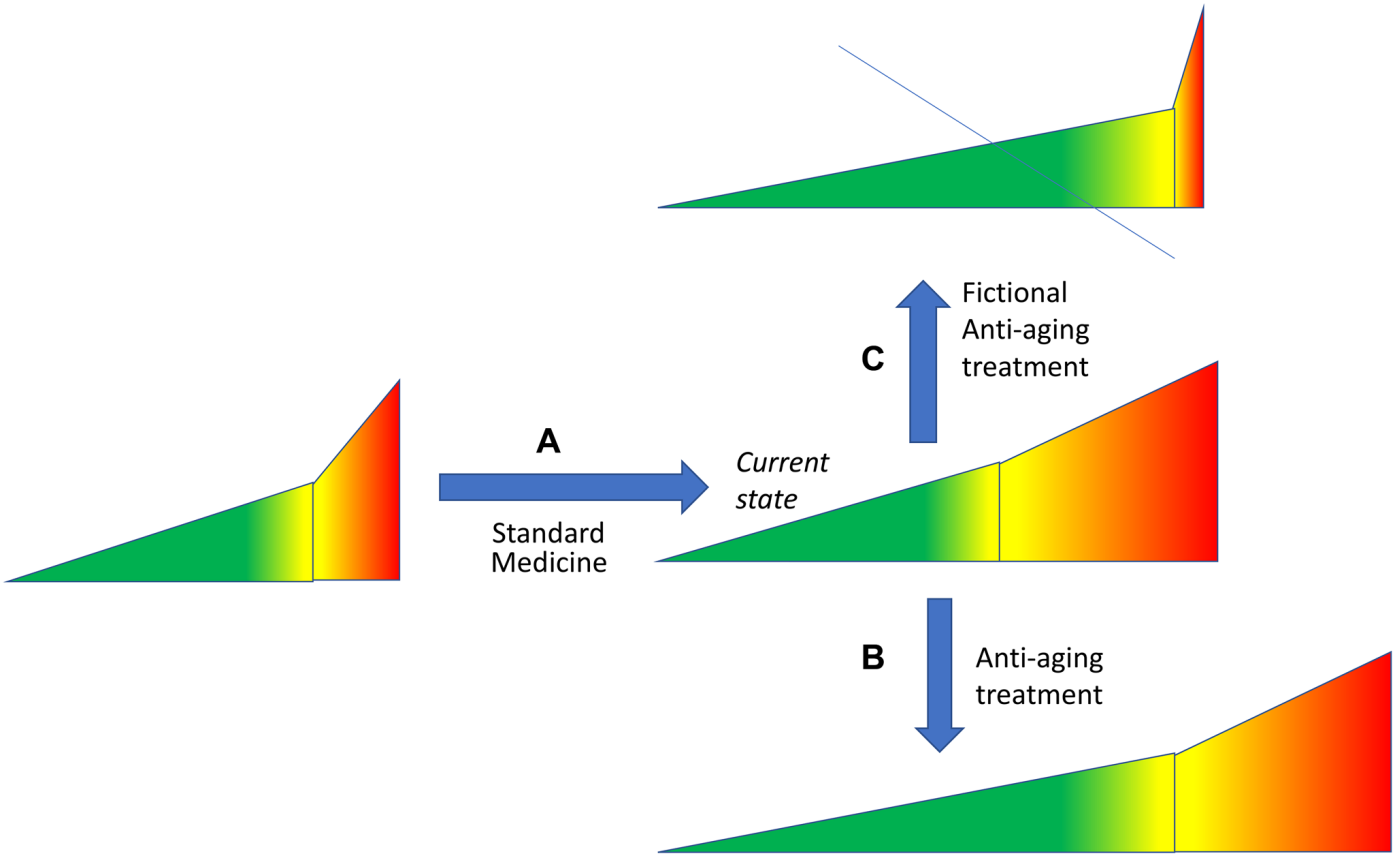
Hypothetical scenarios of adding anti-aging treatments to current medical care. (**A**) Currently, an average lifespan is extended mostly due to health care that mostly extends the morbidity span. (**B**) Anti-aging therapy extends healthspan. If medical care is still available without reduction, the morbidity span would remain the same, and lifespan would be increased further. Morbidity span is relatively decreased (compared to healthspan) but remains constant absolutely. (**C**) Fictional scenario. Introduction of the anti-aging therapy does not extend life. This is only possible if current heath care will be abolished altogether. See Figure 1A for symbols.

Currently, medical interventions extend lifespan mostly by extending morbidity span (Figure 5). For example, insulin therapy may extend lifespan in diabetic patients without reversing the disease. Standard medicine treats each disease individually. In contrast, anti-aging intervention is expected to slow down progression of all age-related diseases [21, 54–56]. By now several interventions were shown to increase healthspan and lifespan in animals. Hypothetically, these interventions may transform an average person into a slowly aging centenarian.

Rapamycin and everolimus are available to delay age-related diseases and increase health span in pets [57] and humans [52, 56, 58]. Rapamycin-based therapy may include medications such as metformin, aspirin, angiotensin-2 antagonists, PDE5 inhibitors, DHEA, melatonin and several others as well as fasting or low carb diets [58]. In theory, anti-aging therapy may make an average human resemble centenarians, aging slower and developing diseases later. Due to anti-aging treatment, these centenarians will reach 100 in good health, just as genetic centenarians.

These centenarians should seek thorough medical care, according to their lower biological age, not according to their chronological age. This, however, will require the revolution of policies, ethical standards and legal issues to ensure maximum longevity.

## References

[1] Vaupel JW. Biodemography of human ageing. Nature. 2010; 464:536–42. 10.1038/nature08984. 20336136 PMC4010874

[2] Modig K, Andersson T, Vaupel J, Rau R, Ahlbom A. How long do centenarians survive? Life expectancy and maximum lifespan. J Intern Med. 2017; 282:156–63. 10.1111/joim.12627. 28470872

[3] Dong X, Milholland B, Vijg J. Evidence for a limit to human lifespan. Nature. 2016; 538:257–59. 10.1038/nature19793. 27706136 PMC11673931

[4] Vijg J, Le Bourg E. Aging and the Inevitable Limit to Human Life Span. Gerontology. 2017; 63:432–34. 10.1159/000477210. 28511176

[5] Weon BM, Je JH. Theoretical estimation of maximum human lifespan. Biogerontology. 2009; 10:65–71. 10.1007/s10522-008-9156-4. 18560989

[6] Olshansky SJ, Carnes BA. Inconvenient Truths About Human Longevity. J Gerontol A Biol Sci Med Sci. 2019 (Suppl 1); 74:S7–12. 10.1093/gerona/glz098. 31001621

[7] Gavrilova NS, Gavrilov LA. Are We Approaching a Biological Limit to Human Longevity? J Gerontol A Biol Sci Med Sci. 2020; 75:1061–67. 10.1093/gerona/glz164. 31276575 PMC7243589

[8] Blagosklonny MV. The goal of geroscience is life extension. Oncotarget. 2021; 12:131–44. 10.18632/oncotarget.27882. 33613842 PMC7869575

[9] Evans CJ, Ho Y, Daveson BA, Hall S, Higginson IJ, Gao W, and GUIDE_Care project. Place and cause of death in centenarians: a population-based observational study in England, 2001 to 2010. PLoS Med. 2014; 11:e1001653. 10.1371/journal.pmed.1001653. 24892645 PMC4043499

[10] Berzlanovich AM, Keil W, Waldhoer T, Sim E, Fasching P, Fazeny-Dörner B. Do centenarians die healthy? An autopsy study. J Gerontol A Biol Sci Med Sci. 2005; 60:862–65. 10.1093/gerona/60.7.862. 16079208

[11] de la Guardia Y, Gilliat AF, Hellberg J, Rennert P, Cabreiro F, Gems D. Run-on of germline apoptosis promotes gonad senescence in C. elegans. Oncotarget. 2016; 7:39082–96. 10.18632/oncotarget.9681. 27256978 PMC5129915

[12] Ezcurra M, Benedetto A, Sornda T, Gilliat AF, Au C, Zhang Q, van Schelt S, Petrache AL, Wang H, de la Guardia Y, Bar-Nun S, Tyler E, Wakelam MJ, Gems D. C. elegans Eats Its Own Intestine to Make Yolk Leading to Multiple Senescent Pathologies. Curr Biol. 2018; 28:2544–56.e5. 10.1016/j.cub.2018.06.035. 30100339 PMC6108400

[13] Wang H, Zhang Z, Gems D. Monsters in the uterus: teratoma-like tumors in senescent C. elegans result from a parthenogenetic quasi-program. Aging (Albany NY). 2018; 10:1188–89. 10.18632/aging.101486. 29923830 PMC6046241

[14] Wang H, Zhao Y, Ezcurra M, Benedetto A, Gilliat AF, Hellberg J, Ren Z, Galimov ER, Athigapanich T, Girstmair J, Telford MJ, Dolphin CT, Zhang Z, Gems D. A parthenogenetic quasi-program causes teratoma-like tumors during aging in wild-type C. elegans. NPJ Aging Mech Dis. 2018; 4:6. 10.1038/s41514-018-0025-3. 29928508 PMC5998035

[15] Blagosklonny MV. Validation of anti-aging drugs by treating age-related diseases. Aging (Albany NY). 2009; 1:281–88. 10.18632/aging.100034. 20157517 PMC2806014

[16] Pavlidis N, Stanta G, Audisio RA. Cancer prevalence and mortality in centenarians: a systematic review. Crit Rev Oncol Hematol. 2012; 83:145–52. 10.1016/j.critrevonc.2011.09.007. 22024388

[17] Kaeberlein M. How healthy is the healthspan concept? Geroscience. 2018; 40:361–64. 10.1007/s11357-018-0036-9. 30084059 PMC6136295

[18] Estapé T. Cancer in the Elderly: Challenges and Barriers. Asia Pac J Oncol Nurs. 2018; 5:40–42. 10.4103/apjon.apjon_52_17. 29379832 PMC5763438

[19] Hong YM. Atherosclerotic cardiovascular disease beginning in childhood. Korean Circ J. 2010; 40:1–9. 10.4070/kcj.2010.40.1.1. 20111646 PMC2812791

[20] Blagosklonny MV. How to save Medicare: the anti-aging remedy. Aging (Albany NY). 2012; 4:547–52. 10.18632/aging.100479. 22915707 PMC3461342

[21] Gems D. The aging-disease false dichotomy: understanding senescence as pathology. Front Genet. 2015; 6:212. 10.3389/fgene.2015.00212. 26136770 PMC4468941

[22] Granville CA, Warfel N, Tsurutani J, Hollander MC, Robertson M, Fox SD, Veenstra TD, Issaq HJ, Linnoila RI, Dennis PA. Identification of a highly effective rapamycin schedule that markedly reduces the size, multiplicity, and phenotypic progression of tobacco carcinogen-induced murine lung tumors. Clin Cancer Res. 2007; 13:2281–89. 10.1158/1078-0432.CCR-06-2570. 17404113

[23] Blagosklonny MV. Prevention of cancer by inhibiting aging. Cancer Biol Ther. 2008; 7:1520–24. 10.4161/cbt.7.10.6663. 18769112

[24] Anisimov VN, Zabezhinski MA, Popovich IG, Piskunova TS, Semenchenko AV, Tyndyk ML, Yurova MN, Antoch MP, Blagosklonny MV. Rapamycin extends maximal lifespan in cancer-prone mice. Am J Pathol. 2010; 176:2092–97. 10.2353/ajpath.2010.091050. 20363920 PMC2861075

[25] Schnohr P, Nyboe J, Lange P, Jensen G. Longevity and gray hair, baldness, facial wrinkles, and arcus senilis in 13,000 men and women: the Copenhagen City Heart Study. J Gerontol A Biol Sci Med Sci. 1998; 53:M347–50. 10.1093/gerona/53a.5.m347. 9754140

[26] Bulpitt CJ, Antikainen RL, Markowe HL, Shipley MJ. Mortality according to a prior assessment of biological age. Curr Aging Sci. 2009; 2:193–99. 10.2174/1874609810902030193. 20021413

[27] Evert J, Lawler E, Bogan H, Perls T. Morbidity profiles of centenarians: survivors, delayers, and escapers. J Gerontol A Biol Sci Med Sci. 2003; 58:232–37. 10.1093/gerona/58.3.m232. 12634289

[28] Willcox DC, Willcox BJ, Wang NC, He Q, Rosenbaum M, Suzuki M. Life at the extreme limit: phenotypic characteristics of supercentenarians in Okinawa. J Gerontol A Biol Sci Med Sci. 2008; 63:1201–08. 10.1093/gerona/63.11.1201. 19038835

[29] Andersen SL, Sebastiani P, Dworkis DA, Feldman L, Perls TT. Health span approximates life span among many supercentenarians: compression of morbidity at the approximate limit of life span. J Gerontol A Biol Sci Med Sci. 2012; 67:395–405. 10.1093/gerona/glr223. 22219514 PMC3309876

[30] Ismail K, Nussbaum L, Sebastiani P, Andersen S, Perls T, Barzilai N, Milman S. Compression of Morbidity Is Observed Across Cohorts with Exceptional Longevity. J Am Geriatr Soc. 2016; 64:1583–91. 10.1111/jgs.14222. 27377170 PMC4988893

[31] Tindale LC, Salema D, Brooks-Wilson AR. 10-year follow-up of the Super-Seniors Study: compression of morbidity and genetic factors. BMC Geriatr. 2019; 19:58. 10.1186/s12877-019-1080-8. 30819100 PMC6394013

[32] Hazra NC, Rudisill C, Gulliford MC. Determinants of health care costs in the senior elderly: age, comorbidity, impairment, or proximity to death? Eur J Health Econ. 2018; 19:831–42. 10.1007/s10198-017-0926-2. 28856487 PMC6008359

[33] Clerencia-Sierra M, Ioakeim-Skoufa I, Poblador-Plou B, González-Rubio F, Aza-Pascual-Salcedo M, Gimeno-Miguel MMA, Prados-Torres A. Do Centenarians Die Healthier than Younger Elders? A Comparative Epidemiological Study in Spain. J Clin Med. 2020; 9:1563. 10.3390/jcm9051563. 32455809 PMC7291259

[34] Couderc AL, Correard F, Nouguerède E, Berbis J, Rey D, Daumas A, Villani P. Centenarians in nursing homes during the COVID-19 pandemic. Aging (Albany NY). 2021; 13:6247–57. 10.18632/aging.202743. 33653968 PMC7993710

[35] Krishnan E, Fries JF, Kwoh CK. Primary knee and hip arthroplasty among nonagenarians and centenarians in the United States. Arthritis Rheum. 2007; 57:1038–42. 10.1002/art.22888. 17665474

[36] Imbelloni LE, Lima U, Pedrosa FK. Successful anesthesia and hip surgery in a 107-year-old patient. Am J Case Rep. 2014; 15:308–11. 10.12659/AJCR.889961. 25072535 PMC4116342

[37] Wu XD, Li Y, Liu JC, Huang W, Qiu GX. Never too old for hip arthroplasty: a 111-year-old woman walks out of hospital-a case report and literature review. Ann Transl Med. 2020; 8:253. 10.21037/atm.2020.01.41. 32309400 PMC7154456

[38] Cassidy RJ, Zhang X, Switchenko JM, Patel PR, Shelton JW, Tian S, Nanda RH, Steuer CE, Pillai RN, Owonikoko TK, Ramalingam SS, Fernandez FG, Force SD, et al. Health care disparities among octogenarians and nonagenarians with stage III lung cancer. Cancer. 2018; 124:775–84. 10.1002/cncr.31077. 29315497 PMC5801133

[39] Walter J, Tufman A, Holle R, Schwarzkopf L. “Age matters”-German claims data indicate disparities in lung cancer care between elderly and young patients. PLoS One. 2019; 14:e0217434. 10.1371/journal.pone.0217434. 31188861 PMC6561547

[40] Pham J, Conron M, Wright G, Mitchell P, Ball D, Philip J, Brand M, Zalcberg J, Stirling RG. Excess mortality and undertreatment in elderly lung cancer patients: treatment nihilism in the modern era? ERJ Open Res. 2021; 7:00393-2020. 10.1183/23120541.00393-2020. 34046489 PMC8141829

[41] Sundberg L, Agahi N, Fritzell J, Fors S. Why is the gender gap in life expectancy decreasing? The impact of age- and cause-specific mortality in Sweden 1997-2014. Int J Public Health. 2018; 63:673–81. 10.1007/s00038-018-1097-3. 29654335 PMC6015620

[42] Le Y, Ren J, Shen J, Li T, Zhang CF. The changing gender differences in life expectancy in Chinese cities 2005-2010. PLoS One. 2015; 10:e0123320. 10.1371/journal.pone.0123320. 25875494 PMC4395256

[43] Rosella LC, Calzavara A, Frank JW, Fitzpatrick T, Donnelly PD, Henry D. Narrowing mortality gap between men and women over two decades: a registry-based study in Ontario, Canada. BMJ Open. 2016; 6:e012564. 10.1136/bmjopen-2016-012564. 28186936 PMC5129136

[44] Meslé F. [Gender gap in life expectancy: the reasons for a reduction of female advantage]. Rev Epidemiol Sante Publique. 2004; 52:333–52. 10.1016/s0398-7620(04)99063-3. 15480291

[45] Kiadaliri A. Avoidable deaths in Sweden, 1997–2018: temporal trend and the contribution to the gender gap in life expectancy. BMC Public Health. 2021; 21:519. 10.1186/s12889-021-10567-5. 33731076 PMC7968161

[46] Colchero F, Aburto JM, Archie EA, Boesch C, Breuer T, Campos FA, Collins A, Conde DA, Cords M, Crockford C, Thompson ME, Fedigan LM, Fichtel C, et al. The long lives of primates and the ‘invariant rate of ageing’ hypothesis. Nat Commun. 2021; 12:3666. 10.1038/s41467-021-23894-3. 34135334 PMC8209124

[47] Harrison DE, Strong R, Sharp ZD, Nelson JF, Astle CM, Flurkey K, Nadon NL, Wilkinson JE, Frenkel K, Carter CS, Pahor M, Javors MA, Fernandez E, Miller RA. Rapamycin fed late in life extends lifespan in genetically heterogeneous mice. Nature. 2009; 460:392–95. 10.1038/nature08221. 19587680 PMC2786175

[48] Anisimov VN, Zabezhinski MA, Popovich IG, Piskunova TS, Semenchenko AV, Tyndyk ML, Yurova MN, Rosenfeld SV, Blagosklonny MV. Rapamycin increases lifespan and inhibits spontaneous tumorigenesis in inbred female mice. Cell Cycle. 2011; 10:4230–36. 10.4161/cc.10.24.18486. 22107964

[49] Johnson SC, Yanos ME, Kayser EB, Quintana A, Sangesland M, Castanza A, Uhde L, Hui J, Wall VZ, Gagnidze A, Oh K, Wasko BM, Ramos FJ, et al. mTOR inhibition alleviates mitochondrial disease in a mouse model of Leigh syndrome. Science. 2013; 342:1524–28. 10.1126/science.1244360. 24231806 PMC4055856

[50] Miller RA, Harrison DE, Astle CM, Fernandez E, Flurkey K, Han M, Javors MA, Li X, Nadon NL, Nelson JF, Pletcher S, Salmon AB, Sharp ZD, et al. Rapamycin-mediated lifespan increase in mice is dose and sex dependent and metabolically distinct from dietary restriction. Aging Cell. 2014; 13:468–77. 10.1111/acel.12194. 24341993 PMC4032600

[51] Johnson SC, Yanos ME, Bitto A, Castanza A, Gagnidze A, Gonzalez B, Gupta K, Hui J, Jarvie C, Johnson BM, Letexier N, McCanta L, Sangesland M, et al. Dose-dependent effects of mTOR inhibition on weight and mitochondrial disease in mice. Front Genet. 2015; 6:247. 10.3389/fgene.2015.00247. 26257774 PMC4510413

[52] Kaeberlein M. Rapamycin and ageing: when, for how long, and how much? J Genet Genomics. 2014; 41:459–63. 10.1016/j.jgg.2014.06.009. 25269671 PMC4401992

[53] Pifferi F, Terrien J, Perret M, Epelbaum J, Blanc S, Picq JL, Dhenain M, Aujard F. Promoting healthspan and lifespan with caloric restriction in primates. Commun Biol. 2019; 2:107. 10.1038/s42003-019-0348-z. 30911682 PMC6420603

[54] Blagosklonny MV. Prospective treatment of age-related diseases by slowing down aging. Am J Pathol. 2012; 181:1142–46. 10.1016/j.ajpath.2012.06.024. 22841821

[55] Blagosklonny MV. Increasing healthy lifespan by suppressing aging in our lifetime: preliminary proposal. Cell Cycle. 2010; 9:4788–94. 10.4161/cc.9.24.14360. 21150328

[56] Blagosklonny MV. Aging and immortality: quasi-programmed senescence and its pharmacologic inhibition. Cell Cycle. 2006; 5:2087–102. 10.4161/cc.5.18.3288. 17012837

[57] Urfer SR, Kaeberlein TL, Mailheau S, Bergman PJ, Creevy KE, Promislow DEL, Kaeberlein M. A randomized controlled trial to establish effects of short-term rapamycin treatment in 24 middle-aged companion dogs. Geroscience. 2017; 39:117–27. 10.1007/s11357-017-9972-z. 28374166 PMC5411365

[58] Blagosklonny MV. Rapamycin for longevity: opinion article. Aging (Albany NY). 2019; 11:8048–67. 10.18632/aging.102355. 31586989 PMC6814615

